# Osteogenesis Imperfecta: A Look into the Cerebellum of the *Brtl* Murine Model

**DOI:** 10.1007/s12035-025-05205-9

**Published:** 2025-07-22

**Authors:** Fabrizio De Luca, Roberta Besio, Emma Lugli, Enrico Pelloni, Claudio Casali, Wendy Pérez Franco, Ludovica Gaiaschi, Federica Gola, Margherita Cavallo, Gloria Milanesi, Antonella Forlino, Maria Grazia Bottone

**Affiliations:** 1 Department of Biology and Biotechnology, University of Pavia, 27100 Pavia, Italy; 2 Biochemistry Unit, Department of Molecular Medicine, University of Pavia, Pavia, Italy

**Keywords:** Osteogenesis imperfecta, Brtl mice, Collagen, Cerebellum, In vivo, Oxidative stress

## Abstract

Osteogenesis imperfecta (OI), also known as brittle bone disease, is a rare congenital connective tissue disorder linked to collagen I defects, commonly known for its skeletal implications. However, collagen fibers play several key roles also in the central nervous system (CNS) starting from CNS development, regulating axonal growth, synaptogenesis, and terminal differentiation of Schwann cells. Collagen I plays an active role in defining brain architecture and in the developing peripheral. Furthermore, collagen is associated with astrogliosis and scar formation, processes finely regulated by molecules that are also involved in the regulation of the oxidative stress pathway. The aim of this work was to evaluate possible alterations in the cerebellum of the *Brtl* mouse, a well characterized model of dominant OI, focusing on cerebellar morphology using hematoxylin–eosin and Picrosirius Red staining, both in bright-field and polarized light microscopy. Additionally, ultrastructural alterations in the different cerebellar neuronal populations were evaluated using transmission electron microscopy (TEM), along with the involvement of the oxidative stress pathway by analyzing the expression levels of specific REDOX markers, such as COX4, SOD2, GPX4, and NRF2. Our results clearly demonstrate morphological and ultrastructural changes in the cerebellum of OI mice, along with increased oxidative stress detected in different regions and cell populations of this CNS area, suggesting possible CNS damage driven by the mutated form of collagen I. This work represents the first report of strong morphological and ultrastructural alterations in several cerebellar areas of *Brtl* mice, as well as a direct impact on the oxidative stress pathway.

**Supplementary Information:**

The online version contains supplementary material available at 10.1007/s12035-025-05205-9.

## Introduction

Collagen is the most abundant protein in mammals, making up around 25% of their total protein content. This structural protein is a key component of connective tissues, e.g., in skin, muscles, bones, and cartilage. Nowadays, at least 29 different types of collagen have been identified and classified into three groups based on their ability to form fibrils [1]. Collagen type I is synthesized as procollagen type I, a heterodimeric molecule composed of two proα1 chains and one proα2 chain. These chains are synthesized and self-associate in the rough endoplasmic reticulum (RER) and are targeted for post-translational modifications until fully assembled in the trimeric molecule [2]. Subsequently, procollagen moves to the Golgi apparatus and is released by exocytosis into the extracellular matrix (ECM), and the globular N- and C-domains are proteolytically cleaved by the proteins ADAMTS-2 and BMP1. The resulting mature type I collagen will be integrated into the ECM [3]. Collagen molecules are covalently cross-linked and packed into dense fibrils, granting mechanical and structural properties to virtually all connective tissues [4]. Osteogenesis imperfecta (OI) is a rare genetic disorder characterized by congenital bone fragility. This results from mutations in the genes that are involved in the synthesis of type I collagen, which is the primary component of the bone matrix [5]. The most prevalent mutations leading to this pathological condition are in the *COL1A1* and *COL1A2* genes, which result in a collagen with an aberrant structure that finally compromises bone strength and function [6]. The mutated collagen is retained within the cell and is responsible for triggering endoplasmic reticulum stress, which can lead to increased levels of oxidative stress [7]. This, in turn, may activate a positive feedback mechanism on collagen production. In fact, oxidative stress can promote the expression of ECM genes, including *COL1A1*, and excessive reactive oxygen species (ROS) production might exacerbate OI symptomatology; e.g., ECM remodeling process is an event strongly affected by oxidative stress and directly linked to the redox damages [8].

The clinical features of OI are highly heterogeneous, ranging from mild forms with only occasional fractures to severe forms, which may include multiple fractures at birth, severe skeletal deformities and growth delay, and lethal forms [9]. Therefore, the mobility of OI patients is frequently impaired by recurrent fractures and subsequent immobilization, which can result in muscle wasting and joint stiffness [10]. In addition to the musculoskeletal alterations, emerging research suggests that the nervous system may also play a role in the disease pathology. Some studies have reported that collagen mutations, affecting bone strength, can also impact the nervous system, potentially leading to issues such as altered pain perception and neuromuscular dysfunction [11]. Moreover, there is evidence that collagen fibers are crucial for the proper functioning of neurons, and its deficiency may result in synaptic dysfunction and related impairment of neural connectivity [12]. In particular, microarray analysis during development has shown that type I collagen may be involved in cerebellar development, guiding neuronal outgrowth, maturation, circuit formation, and synaptogenesis, in collaboration with other components of the extracellular matrix (ECM), given its transient expression in the developing cerebellum, which tends to decrease during the third postnatal week, when this CNS area reaches maturity [13, 14]. Moreover, abnormalities in the neurocranial skeleton may reflect on the underlying nervous system and reverberate on brain morphology, leading to cerebellar displacement and cerebellar hypoplasia, as well as neurologic abnormalities of both the brain and spinal cord, as reported in several OI cases, corroborating the negative impact of this pathological condition in the central nervous system of OI patients [15, 16].

Animal models encompassing the disorder are available, comprehending different clinical phenotypes of OI, providing the opportunity to assess the geometric and biomechanical characteristics of bones with sufficient statistical reliability. The heterozygous *Col1a1*^+/G349C^ mouse, named *Brtl*, was the first murine OI knock-in model to exhibit non-lethal characteristics. The mutation, α1(I)-Gly349Cys, was initially identified in two probands with moderately severe OI and subsequently in one lethal case [17].

Based on literature data, indicating that the synthesis and stability of various collagens, including type I collagen, are finely regulated by oxidative stress in different body tissues, ranging from skin to ligaments, cardiac tissue, and the kidney [18–22], and given the well-documented presence and distribution of type I collagen in the CNS [23], along with scientific studies highlighting the involvement of collagen mutations in chronic pain, coordination issues and overall motor function impairment, the proposed work aims to investigate the possible alterations/changes in the cerebellum of Brtl *mice*, focusing on the involvement of mutated collagen fibers in the cerebellar (i) morphology, (ii) ultrastructural changes, and (iii) modification in the expression levels of specific markers of oxidative stress pathway, to better understand the effects of OI in the central nervous system.

## Materials and Methods

### Mouse Strain and Genotyping

CD1/129 Sv/B6 *Col1a1*^+*/G349C*^ mice (Brtl), carrying in heterozygosity a G349C substitution in the collagen I α1 chain, were used for this study [24]. Mutant mice and control littermates were maintained under standard experimental animal care protocol following Italian Laws in the centralized animal facility of the University of Pavia, Italy. All the experiments were approved by the Office for the Animals Welfare of the University of Pavia and by the Italian Ministry of Health (protocol n. 243/2018-PR, 27/03/18), in accordance with the EU Directive 2010/63/EU. Genomic DNA was extracted from tail clip and genotyping was performed by PCR as previously reported [25].

### Necropsy and Cerebellar Specimen Preparation

Eighteen-month-old mice (5 animals for each experimental group) were sacrificed as previously reported [26, 27]. Brains were immediately removed and rapidly fixed for 48 h in 4% paraformaldehyde—0.1 M phosphate buffer pH 7.4, subsequently dehydrated in ethanol followed by immersion in acetone, and finally embedded in Paraplast X-TRA (Sigma-Aldrich, Milan, Italy). Eight-micrometer-thick cerebellar sections were cut in the sagittal plane and collected on silane-coated slides.

### Hematoxylin and Eosin Histochemistry

The histochemical and immunohistochemical evaluations focused on the cerebellum.

To evaluate the gross morphology and neuronal cytoarchitecture of (i) different cerebellar cortical layers, i.e., molecular layer (ML), Purkinje cell layer (PC), and internal granular layer (IGL), (ii) deep cerebellar nuclei, and (iii) choroid plexus by light microscopy, hematoxylin and eosin (H&E) staining was performed as previously reported [28].

Briefly, approximately 20 randomized Sects. (5 microscopic fields) per animal and experimental condition were examined by a blinded operator using a Leica DM6B WF microscope (Leica Microsystems, Buccinasco, MI, Italy). The images were acquired with Leica dfc 7000 t CCD camera (Leica microsystems, Buccinasco, MI, Italy) and stored using a Leica Application Suite X (LAS X) software (Version 5.1.0).

### Picrosirius Red Staining

Sagittal sections of the cerebellum were stained using a solution of Picrosirius Red (PSR) (0.1% Sirius Red prepared in saturated aqueous picric acid) for 1 h. Subsequently, the samples were rinsed in 5% acidic water for staining of collagen bundles [29]. Finally, sections were dehydrated in ethanol, immersed in xylene, and lastly mounted in Eukitt (Kindler, Freiburg, Germany). Images in bright fields and polarized light were acquired using a Leica DM6B WF microscope (Leica Microsystems, Buccinasco, MI, Italy), equipped with Leica dfc 7000 t CCD camera (Leica microsystems, Buccinasco, MI, Italy) and stored using a Leica Application Suite X (LAS X) software (Version 5.1.0). The labeling intensity, measured as optical density (OD), was determined through densitometric analysis, following previous literature methods [26–29]. To avoid possible variations in the measured birefringence, histological samples were serially sectioned, and the sections were mounted on slides while maintaining a consistent orientation of the sample on the glass slide. During image acquisition using polarized light microscopy, the optimal angle was maintained for each sample. Additionally, other variable factors were controlled, including variations in light source intensity and exposure time, as well as non-uniform field exposure in the captured images. The sample tissue area and section thickness were also kept constant, ensuring consistency across all analyses [30].

### Immunohistochemistry and Quantitative Analysis

The immunohistochemical assessment and subsequent quantitative evaluations were performed as previously published [31]. Briefly, commercial antibodies were used to perform immunohistochemical reactions on mouse cerebellar samples to evaluate the distribution and expression levels of specific markers (listed in Table 1).

**Table 1:** Primary and secondary antibodies employed for immunohistochemical stains

	**Antigen**	**Manufacturer, species,** **mono-polyclonal, cat./lot**	**Dilution**
***Primary*** ***antibodies***	Anti-COX4 (20E8C12)	Abcam (Cambridge, MA, USA), mouse monoclonal IgG2a, cat# ab14744, RRID:AB_301443	1200
	Anti-SOD2 (D3X8F)	Cell Signaling Technology (Danvers, MA, USA), rabbit monoclonal IgG, cat# 13,141, RRID:AB_2636921	1–200
	GPX4	Abcam (Cambridge, MA, USA), rabbit polyclonal IgG, cat# ab231174, RRID:AB_3073732	1:100
	NRF2	Abcam (Cambridge, MA, USA), rabbit polyclonal IgG, cat# ab31163, RRID:AB_881705	1–200
***Secondary*** ***antibodies***	Biotinylated horse anti-mouse IgG	Vector Laboratories (Burlingame, CA, USA), horse, cat# PK-6102, RRID:AB_2336821	1:200
	Biotinylated goat anti-rabbit IgG	Vector Laboratories (Burlingame, CA, USA), goat, cat# PK-6101, RRID:AB_2336820	1:200

Appropriate biotinylated secondary antibodies (listed in Table 1) and an avidin biotinylated horseradish peroxidase complex (Vector Laboratories, Burlingame, CA, USA) were utilized to identify antigen sites. 3,3′-diaminobenzidine tetrahydrochloride peroxidase substrate was employed as the chromogen (Sigma-Aldrich, St. Louis, MO, USA). Nuclei were counterstained using Carazzi’s hematoxylin. Finally, samples were dehydrated in ethanol, cleared with xylene, and mounted in Eukitt (Kindler, Freiburg, Germany). Immunostained sections were observed with a Leica DM6B WF microscope (Leica microsystems, Buccinasco, MI, Italy), and images were acquired with a Leica dfc 7000 t CCD camera (Leica microsystems, Buccinasco, MI, Italy), and results were analyzed using the Leica Application Suite X (LAS X) software (Version 5.1.0). The magnification reported in the caption of each figure refers solely to the magnification of the objectives used. To avoid potential differences in results linked to slight procedural variations, all immunostaining reactions were performed simultaneously on slides from different experimental groups. As a negative control, some sections were incubated using PBS (without primary antibodies); no immunoreactivity was observed under this condition. As a further control, non-immune antibodies from the same animal species as the primary antibody were used for each marker investigated. The extent of histochemical and immunohistochemical labeling was evaluated on section images acquired from exposure times that avoid any pixel saturation. The immunolabeling intensity, measured as optical density (OD), was determined through densitometric analysis, following previous literature methods [26–29]. In particular, the OD was evaluated in 3 randomly chosen images/sections, with at least 10 measurements performed per image for 5 photographs/animal in each experimental group. The following evaluations were also conducted: count of cell density (number of cells/mm^2^) and immunopositive cell density (number of immunopositive cells/mm^2^). The results were reported as the average of the values measured in individual animals (5 animal/group). Data were recorded using Excel software, and the analysis was conducted using ImageJ 1.48i software (NIH, Bethesda, MA, USA).

### Transmission Electron Microscopy Evaluations

Control and *Brtl* cerebellar specimens were processed for TEM analysis as previously described [32]. Briefly, cerebellar tissues (small samples of cerebellar vermis of about 1 mm^3^) were fixed for 2 h by immersion in 1.5% glutaraldehyde (Polysciences, Inc., Warrington, PA, USA) buffered with 0.07 M cacodylate buffer (pH 7.4), containing 7% sucrose, followed by post-fixation in OsO_4_ (Sigma Chemical Co., St. Louis, MO, USA) in 0.1 M cacodylate buffer (pH 7.4) for 2 h at 4 °C, dehydrated in increasing concentrations of acetone, and finally embedded in epoxy resin. Subsequently, ultrathin Sects. (70–80 nm) were cut on a Reichert OM-U3 ultramicrotome, collected on nickel grids, and stained with lead citrate. Lastly, sections were observed under a JEM 1200 EX II (JEOL, Peabody, MA, USA) electron microscope, equipped with a MegaView G2 CCD camera (Olympus OSIS, Tokyo, Japan) and operating at 100 kV, and then processed using the iTEM software (version n. v.5.1). ER thickness and mitochondria area were evaluated in 3 randomly chosen images/sections, with at least 10 measurements performed per image for 5 photographs/animal in each experimental group. Only granule cells from the IGL were selected for the electron microscopy analysis of mitochondrial area (µm^2^) and ER lumen width (nm). Data were recorded using Excel software, and the analysis was conducted using ImageJ 1.48i software (NIH, Bethesda, MA, USA).

### Statistics

Histochemical and immunohistochemical data were expressed as mean ± SEM. The Anderson–Darling, D’Agostino & Pearson, Shapiro–Wilk, and Kolmogorov–Smirnov tests were performed to study the normality distributions of data. Subsequently, results were analyzed to verify statistically significant differences. In particular, for normally distributed data, the analysis was performed employing an unpaired *t*-test. Diversely, for results that did not pass the normality test, the analyses were conducted using the Mann–Whitney test. The differences were considered statistically significant for *p* < 0.05 (*), *p* < 0.01 (**), and *p* < 0.001 (***).

## Results

### Cerebellar Cytoarchitecture Alteration

**Fig. 1:**
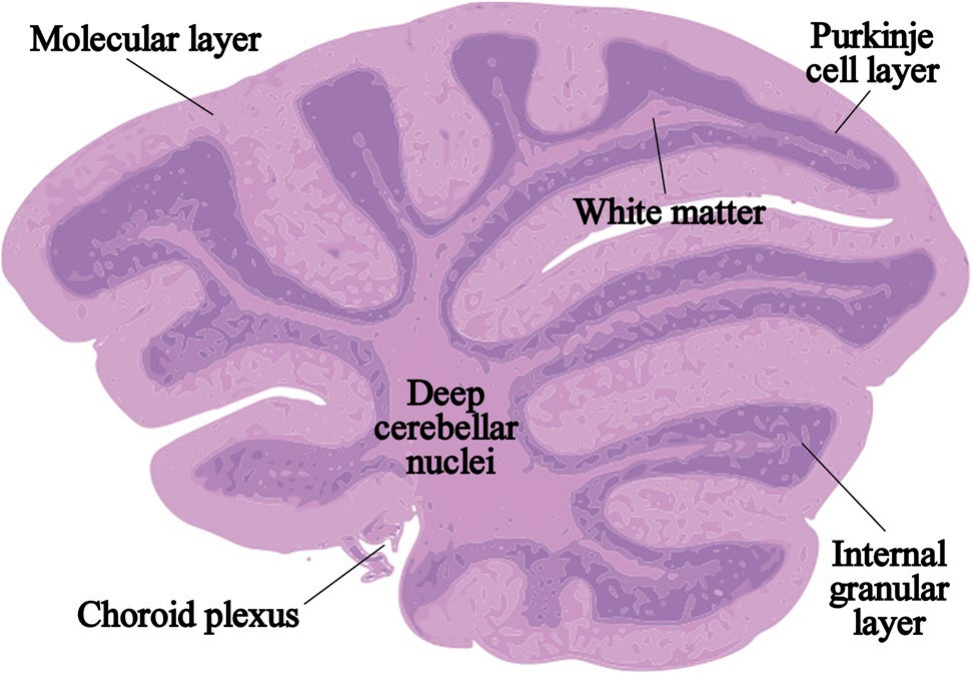
Graphical representations of mouse cerebellar section in the sagittal plane, showing the cortical and deeper investigated areas

To assess the possible alteration/changes in the cytoarchitecture of the cerebellum in 18-m-old male and female Brtl mice, H&E staining was carried out. The cerebellar cortex was divided into three areas—molecular layer (ML), Purkinje cell layer (PCL), and internal granular layer (IGL). Parallelly, in the deep portion of the cerebellum, we find the white matter (WM), the deep cerebellar nuclei (DCN), and, in the fourth ventricle, the choroid plexus (CP) (Fig. 1).

**Fig. 2:**
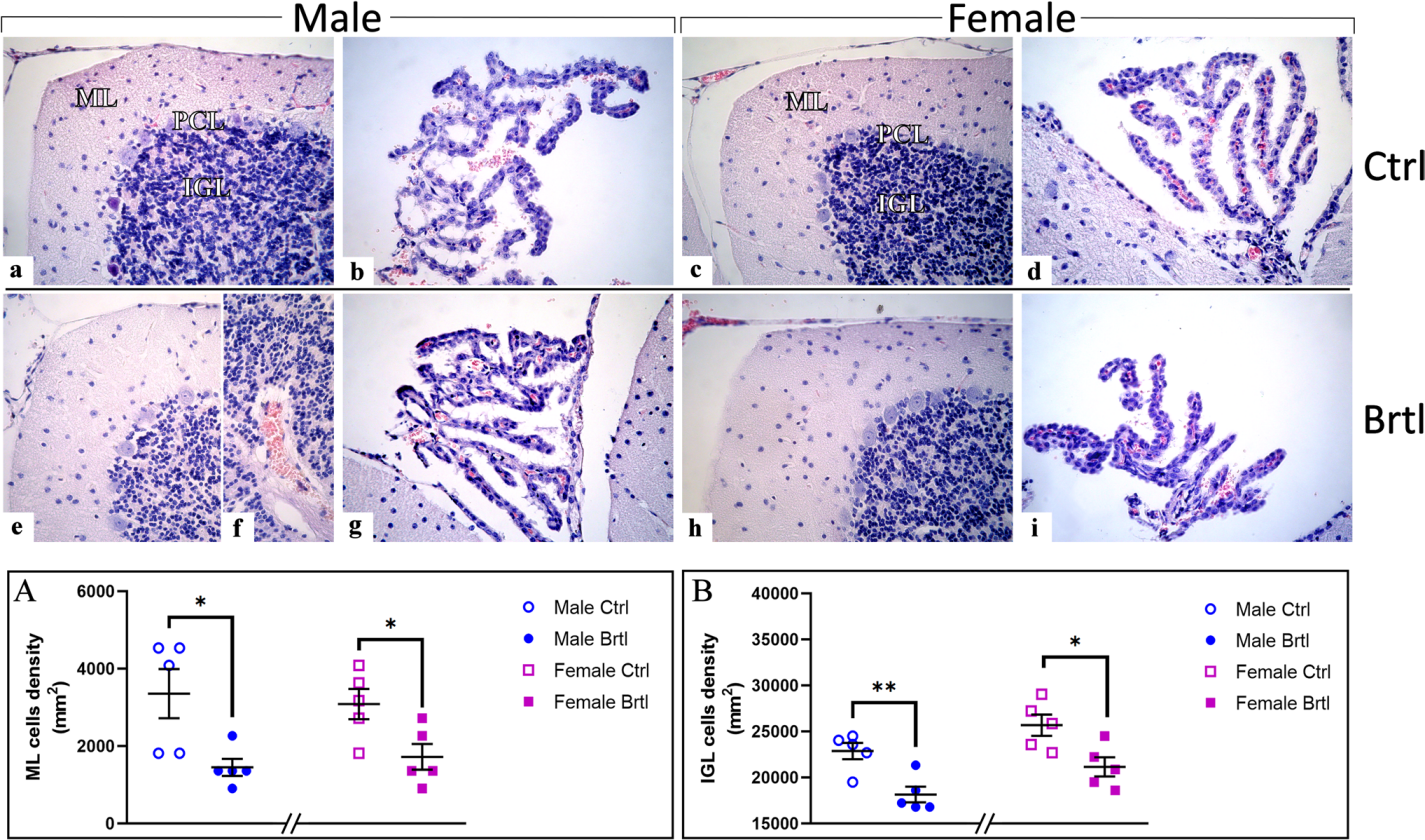
H&E staining showing the well-preserved physiological cerebellar cytoarchitecture in male (**a**) and female (**c**) controls and *Brtl* mice (**e**, **f**, and **h **for male and female, respectively). **a**, **c**, **e**, and **h**: Low magnification micrograph shows the cerebellar cortex (subdivided into ML, PCL, and IGL) and (**b**, **d**, **g**, and **i**) the CP. **f**: Higher magnifications of the IGL area displaying hemorrhagic foci. Light microscopy magnification: × 40 (**a**–**e**, **g**–**i**); × 60 (**f**). Histograms display the quantitative valuation of ML (Panel **A**) and IGL (Panel **B**) cell density. *p*-values: (*) *p* < 0.05 and (**) *p* < 0.01

The overall general morphology of the cerebellar was conserved in both genders of control and *Brtl* animals. In particular, all experimental groups exhibited the characteristic three layers in the cerebellar cortex, i.e., ML, PCL, and IGL, and a physiological morphology of CP. However, a significant reduction of ML and IGL cell density was observed in both male and female *Brtl* mice, compared to control animals (Fig. 2, Panel A, Table S1, and Panel B, Table S2, for ML cell density and IGL cell density respectively). Moreover, several hemorrhagic foci widely distributed in the thickness of IGL were observed only in the cerebellar cortex of male *Brtl* mice. Furthermore, it should be noted that there is a slight increase in cell density measured in the IGL of the control female mice compared to the same layer in the control male samples (data not shown).

### PSR Staining: Collagen Network Changes

**Fig. 3:**
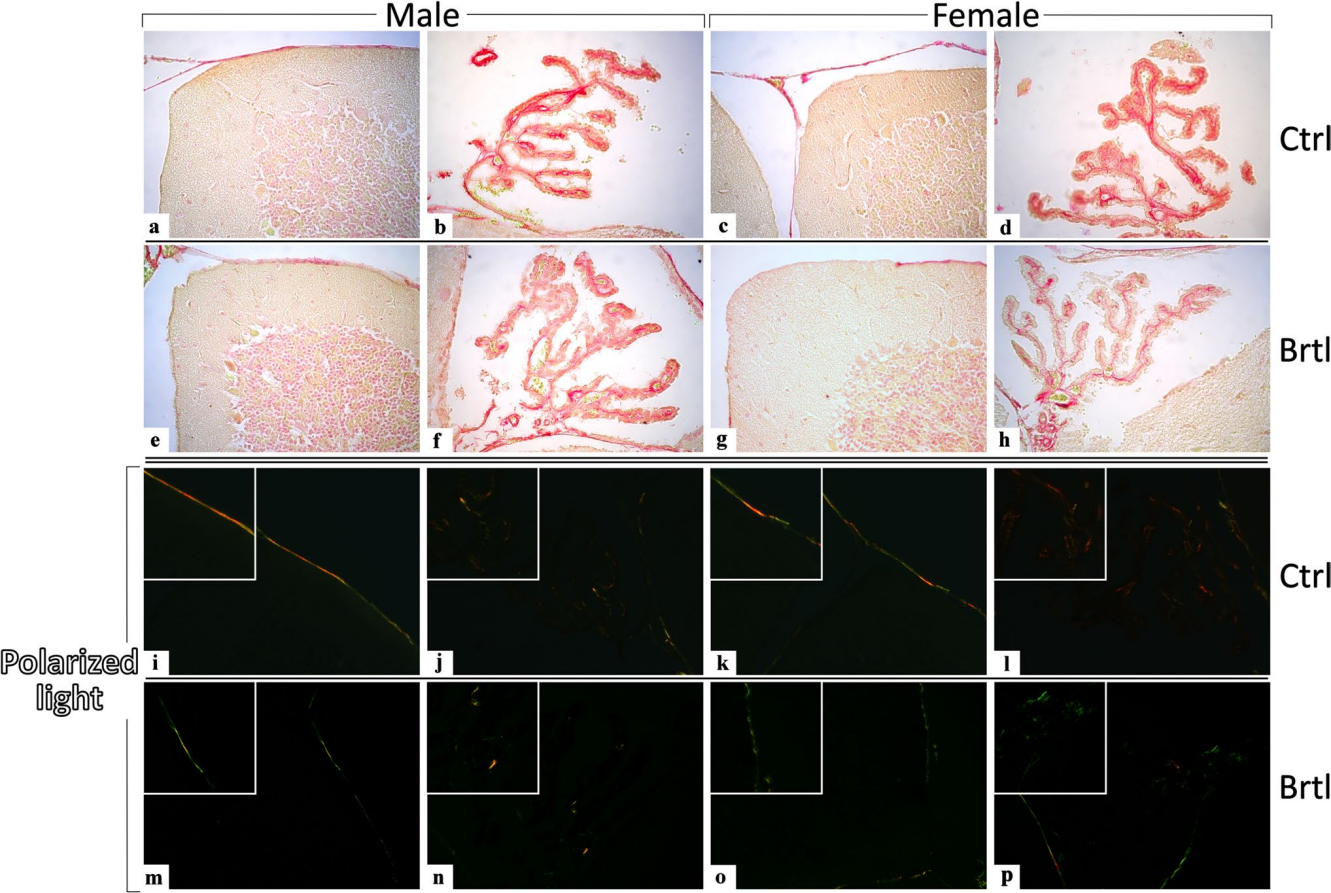
PSR staining evaluation under bright-field (**a**–**h**) and polarized light (**i**–**p**) microscopy. Polarized light allows the discrimination between thick (red signals) and thin (green signals) collagen fibrils. Representative cerebellar specimens showing cerebellar cortex (**a**, **c**, **e**, **g**, **i**, **k**, **m**, and **o**) and CP (**b**, **d**, **f**, **h**, **j**, **l**, **n**, and **p**) from control (**a**, **b**, **i**, **j** and **c**, **d**, **k**, **l** for male and female respectively) and *Brtl* animals (**e**, **f**, **m**, **n** and **g**, **h**, **o**, **p** for male and female respectively). Magnification: × 40; inserts: × 60

**Fig. 4:**
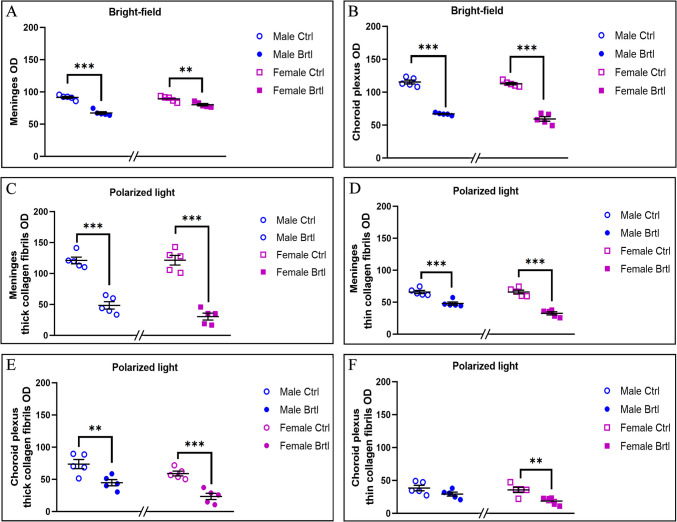
Histograms showing PSR OD measurement in bright-field (Panels **A** and **B**) and polarized light (Panels **C**, **D**, **E**, and **F**). Panel (**A**) and Panel (**B**): bright-field PSR OD measurements in meninges and CP, respectively. Panel(**C**) and Panel (**D**): polarized light PSR analyses of meninges showing the thick and thin collagen OD measurements, respectively. Panel (**E**) and Panel (**F**): polarized light PSR evaluation of CP showing the thick and thin collagen OD data respectively. *p*-values: (**) *p* < 0.01 and (***) *p* < 0.001

To evaluate the collagen fibrils organization, Picrosirius Red staining (PSR) was employed [26]. Bright-field microscopy revealed a strong Sirius Red positivity in the meninges and CP of all experimental groups (Fig. 3, a–h). Moreover, the quantitative analyses of bright field PSR positive OD revealed an extremely significant reduction of labeling in both the meninges and CP of *Brtl* animals, with a similar extent in male and female mice (Fig. 4, Panel A, Table S3, and Panel B, Table S4, for meninges OD and CP OD respectively). To better discriminate between the expression level of thick and thin collagen fibrils, the PSR stain was subsequently observed using polarized light microscopy [33, 34] (Fig. 3, i–p). Thick and thin collagen fibrils appear well-defined in both meninges and CP of control and *Brtl* mice. However, the quantitative analyses of OD highlighted an extremely significant reduction of labeled thick collagen in the meninges and CP of both male and female *Brtl* mice, compared to control (Fig. 4, Panel C, Table S5, and Panel E, Table S7, for meninges thick collagen OD and CP thick collagen OD respectively). Similarly, an extremely significant reduction of meninges thin collagen OD was detected in both male and female *Brtl* groups, compared to control (Fig. 4, Panel D, Table S6). Parallelly, a very significant reduction of thin collagen OD was observed in the CP of female *Brtl* mice and only a slight decrease in the male *Brtl* group, compared to the control condition (Fig. 4, Panel F, Table S8).

### Oxidative Stress Pathway Activation

To evaluate the possible changes of redox state in the cerebellar tissue of Brtl mice, the expression levels of specific markers of oxidative stress pathway were analyzed, i.e., cyclooxygenase 4 (COX4), superoxide dismutase 2 (SOD2), nuclear factor erythroid 2–related factor 2 (Nrf2), and glutathione peroxidase 4 (GPX4).

**Fig. 5:**
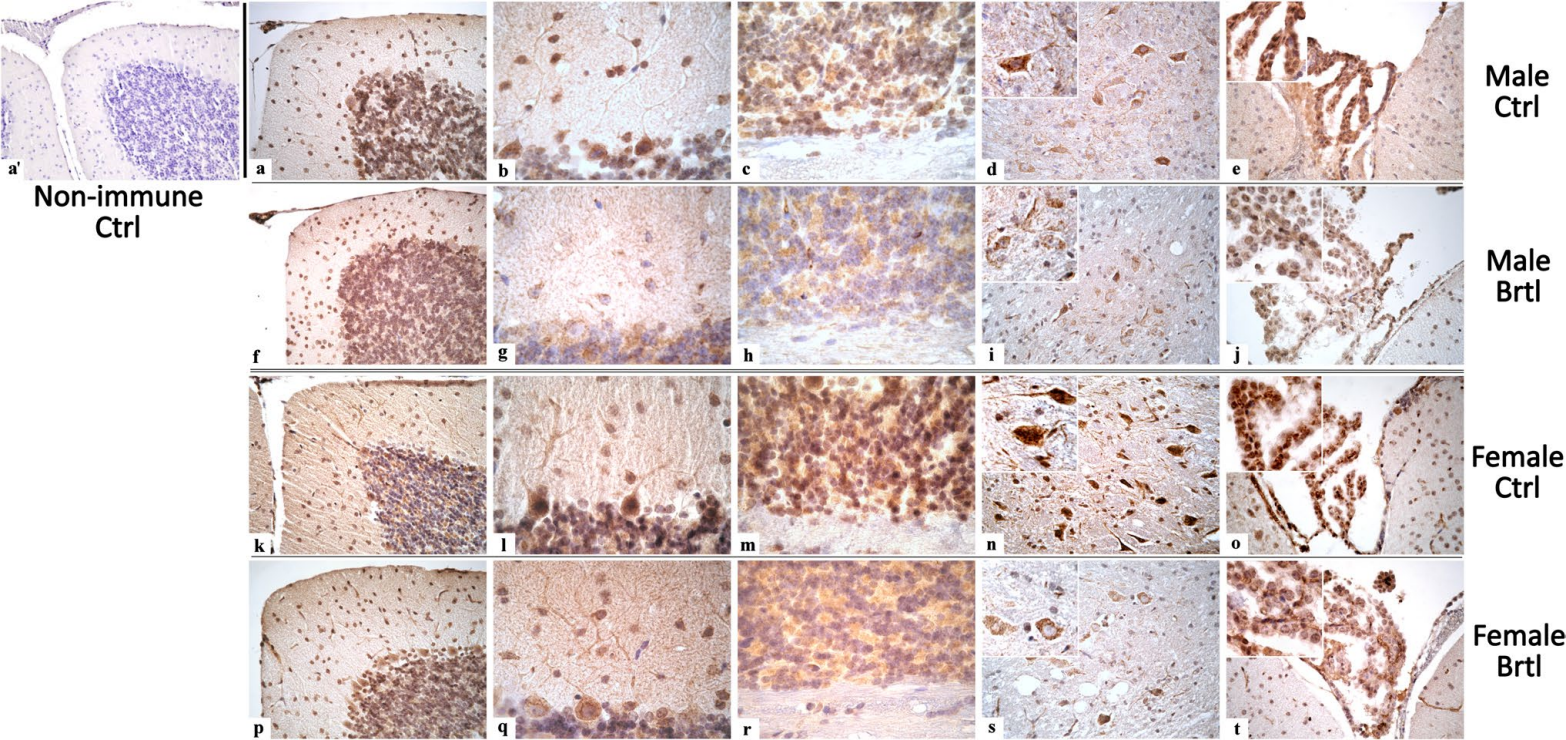
Micrographs depicting immunohistochemical reaction for COX4 in the cerebellum from control (**a**–**c** and **k**–**o** for male and female respectively) and Brtl animals (**f**–**j** and **p**–**t** for male and female respectively). Non-immune control (**a’**). Magnification: × 40 (**a’**, **a**, **d**, **e**, **f**, **i**, **j**, **k**, **n**, **o**, **p**, **s**, and **t**); × 60 (**b**, **c**, **g**, **h**, **l**, **m**, **q**, **r**, and inserts)

**Fig. 6:**
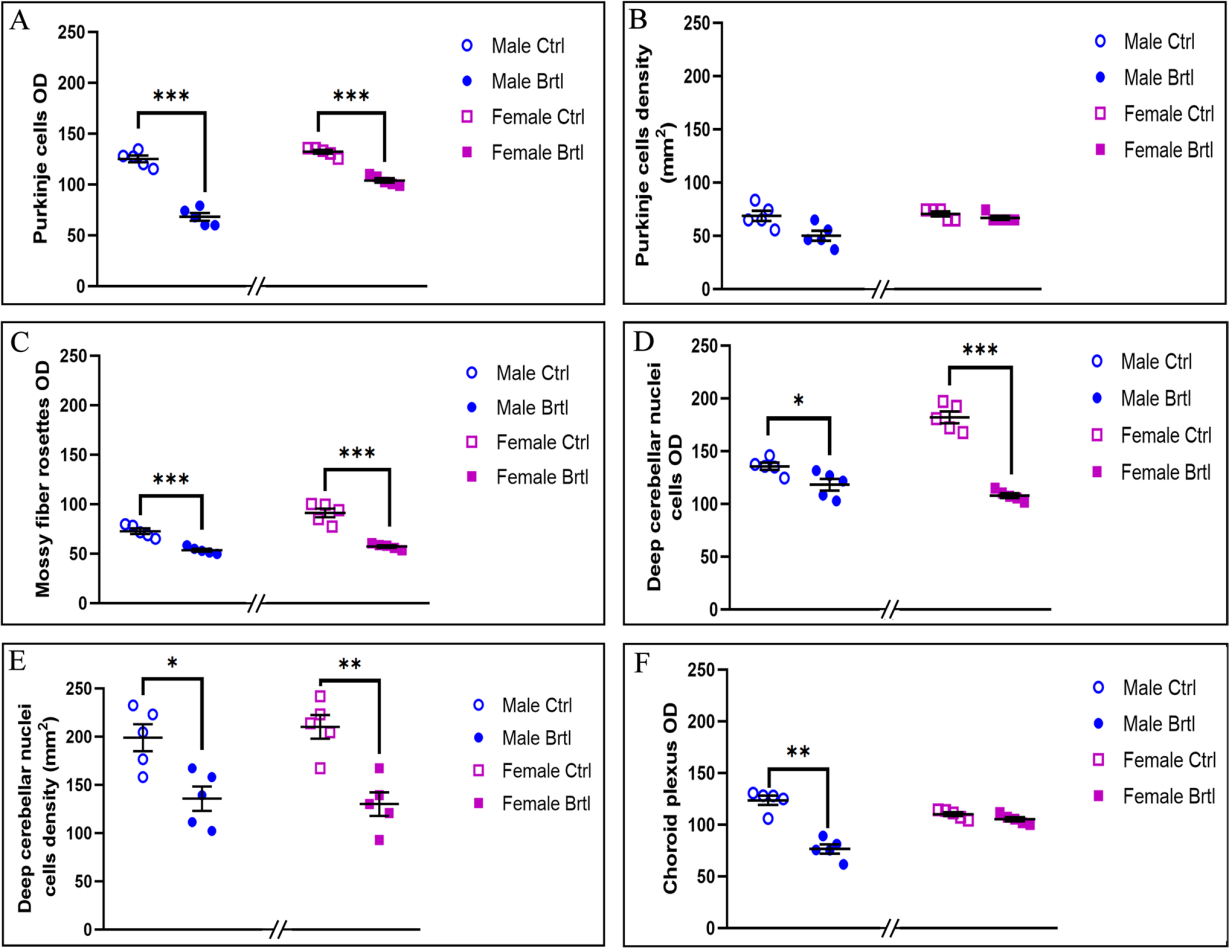
Histograms illustrating COX4-immunopositive OD (Panels **A**, **C**, **D**, and **F**) and cell density (Panels **B** and **E**) assessed in cerebellar layers and CP of control and Brtl mice. *p*-values: (*) *p* < 0.05, (**) *p* < 0.01, and (***) *p* < 0.001

Immunofluorescence reaction for COX4, an enzyme involved in the mitochondrial respiratory chain, facilitating the transfer of electrons from cytochrome c to oxygen [35], revealed a strong and widespread immunopositivity for this enzyme in the soma and dendritic tree of Purkinje cells, as well as in mossy fibers rosettes of the IGL, in the cells of DCN and endothelial cells of CP (Fig. 5). Quantitative analyses revealed an extremely significant decrease of COX immunopositive OD in the Purkinje cells of both male and female *Brtl* mice, compared to control groups (Fig. 6, Panel A, Table S9), despite the OD values measured in the mutated female group remaining higher than those detected in the same group of male mice (data not shown). Differently, only a slight but not significant decrease of COX4 immunopositive Purkinje cell density was observed in *Brtl* groups, more pronounced in males than in females mice (Fig. 6, Panel B, Table S10). An extremely significant decrease of COX4 immunopositive OD was also observed in the IGL mossy fiber rosettes of *Brtl* mice compared to control, with a similar extent in male and female groups (Fig. 6, Panel C, Table S11). It should be noted that the immunopositivity for this mitochondrial enzyme measured in the IGL mossy fiber rosettes was slightly higher in control female mice compared to control male animals. Similarly, the COX4 immunopositive OD in the deep cerebellar nuclei under physiological conditions was also greater in female mice compared to male mice (data not shown). However, a significant reduction in both COX4 immunolabeled OD and COX4 immunopositive cell density was visible in the cerebellar nuclei of both male and female Brtl mice when compared to their respective control groups (Fig. 6, Panel D, Table S12, and Panel E, Table S13, for COX4 immunopositive OD and cell density respectively). Conversely, the analysis of COX4 OD in the CP showed a very significant reduction only when comparing the male groups. No difference was observed in the COX4 OD between the female experimental groups (Fig. 6, Panel F, Table S14).

**Fig. 7:**
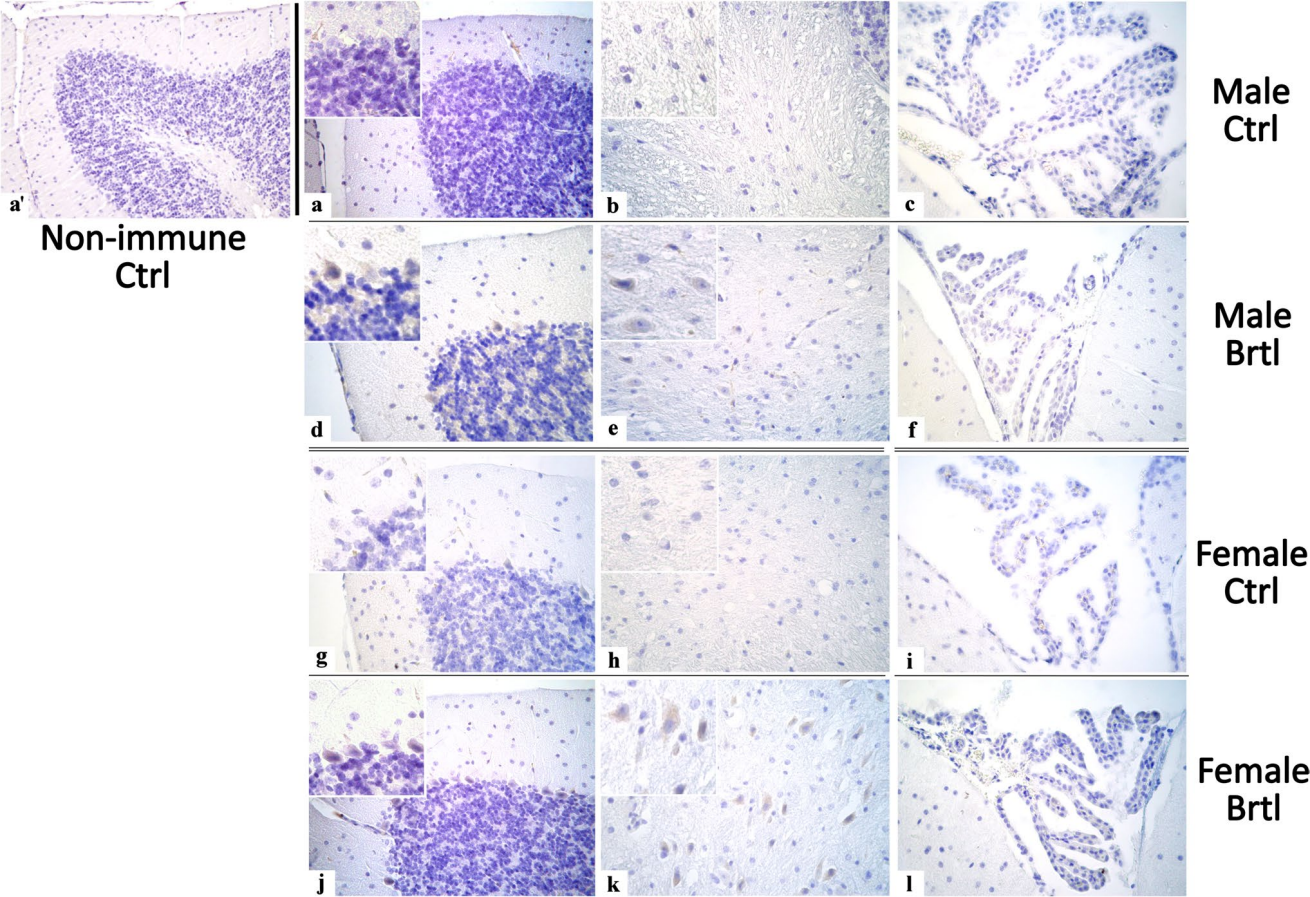
Micrographs depicting immunohistochemical reaction for SOD2 in the cerebellum from control (**a**–**c** and **g**–**i** for male and female respectively) and Brtl animals (**d**–**f** and **j**–**l** for male and female respectively). Non-immune control (**a’**). Magnification: × 40 (**a’** and **a**–**l**); × 60 (inserts)

The immunopositivity for the subsequent oxidative stress markers evaluated, i.e., SOD2, GPX4, and NRF2, was localized only in the soma of Purkinje cells, as well as in the IGL mossy fibers rosettes, DCN cells, and CP endothelial cells (Fig. 7, Fig. 9, and Fig. 11 for SOD2, GPX4, and NRF2 respectively).

**Fig. 8:**
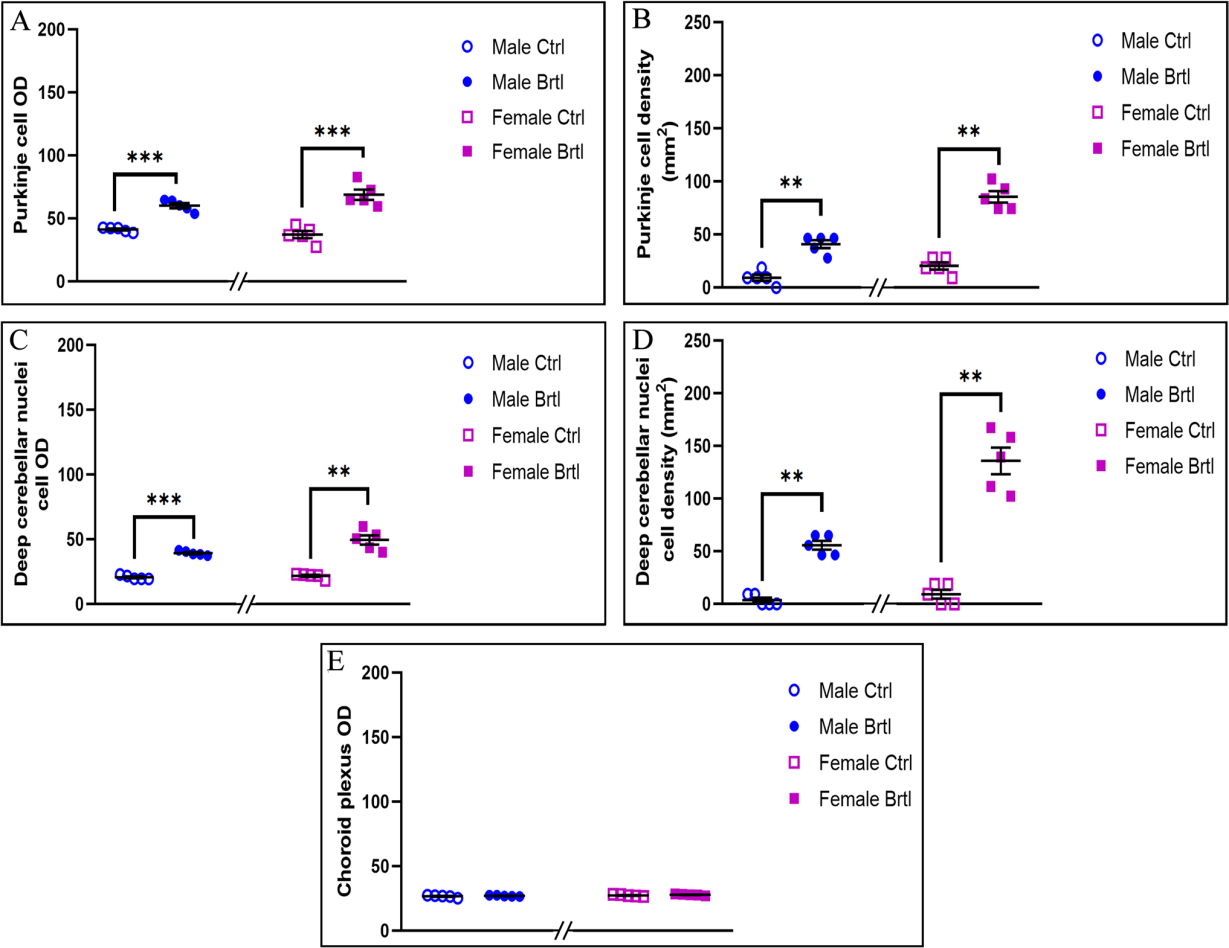
Histograms illustrating SOD2-immunopositive OD (Panels **A**, **C**, and **E**) and cell density (Panels **B** and **D**) assessed in cerebellar layers and CP of control and Brtl mice. *p*-values: (**) *p* < 0.01 and (***) *p* < 0.001

To further investigate the activation of the oxidative stress pathway in the central nervous system of *Brtl* mice, immunoreaction for SOD2, a key player in the defense against free radicals productions [35], was employed (Fig. 7). The immunostaining for SOD2 revealed a significant increase in the immunopositive OD and cell density in both Purkinje cells (Fig. 8, Panel A, Table S15, and Panel B, Table S16, for SOD2 immunopositive Purkinje cell OD and cell density, respectively) and deep cerebellar nuclei cells (Fig. 8, Panel C, Table S17, and Panel D, Table S18, for SOD2 immunopositive DCN cell OD and cell density, respectively) of *Brtl* animals of both sexes compared to the control groups. Interestingly, the optical density values and the density of immunostained cells for this enzyme in the aforementioned areas were slightly higher in female *Brtl* animals compared to male Brtl mice (data not shown). No significant difference was observed when evaluating the SOD2 immunopositive OD of the endothelial cells in the CP across the different experimental groups considered (Fig. 8, Panel E, Table S19).

**Fig. 9:**
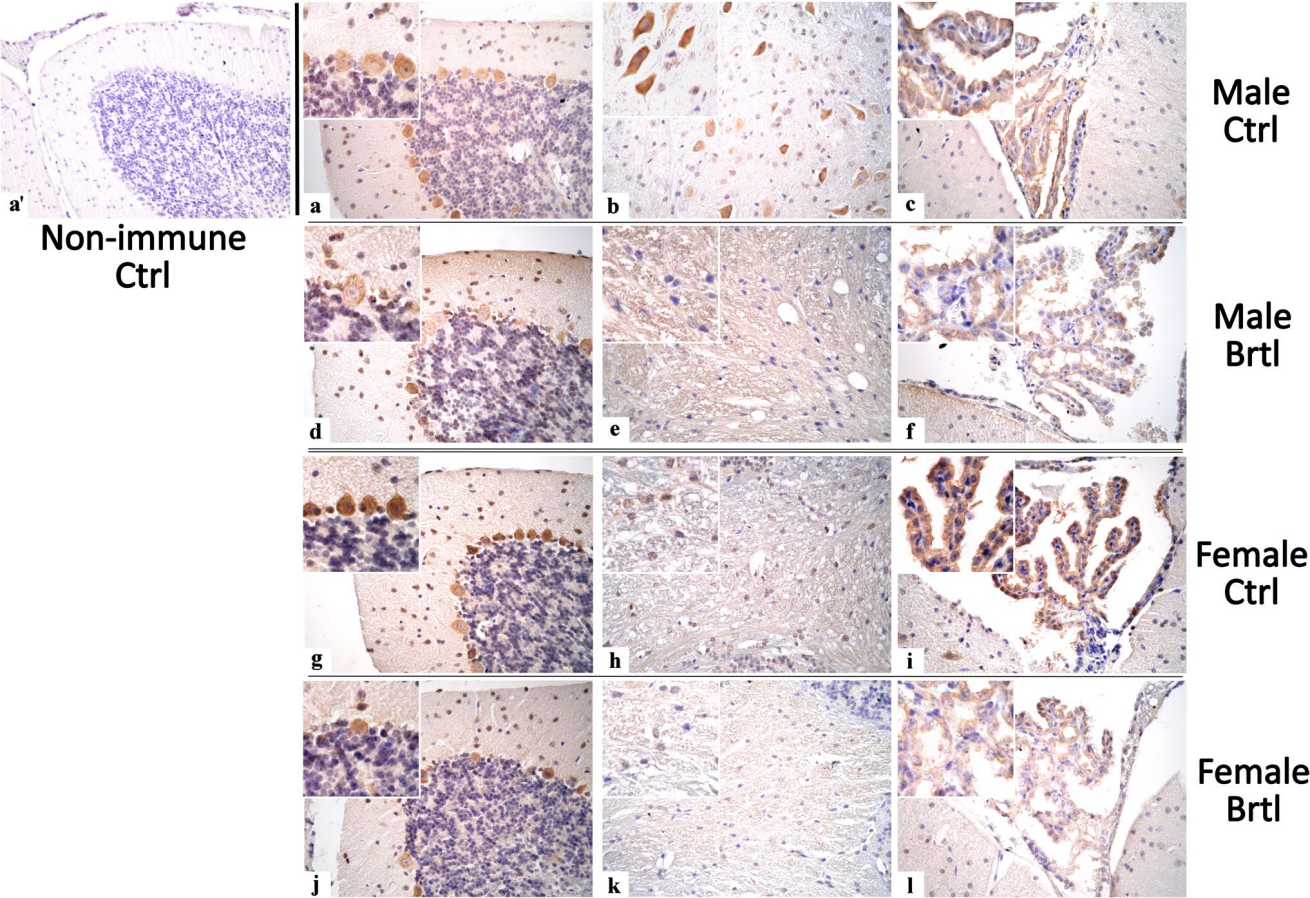
Micrographs depicting immunohistochemical reaction for GPX4 in the cerebellum from control (**a**–**c** and **g**–**i** for male and female respectively) and Brtl animals (**d**–**f** and **j**–**l** for male and female respectively). Non-immune control (**a’**). Magnification: × 40 (**a’** and **a**-**l**); × 60 (inserts)

**Fig. 10:**
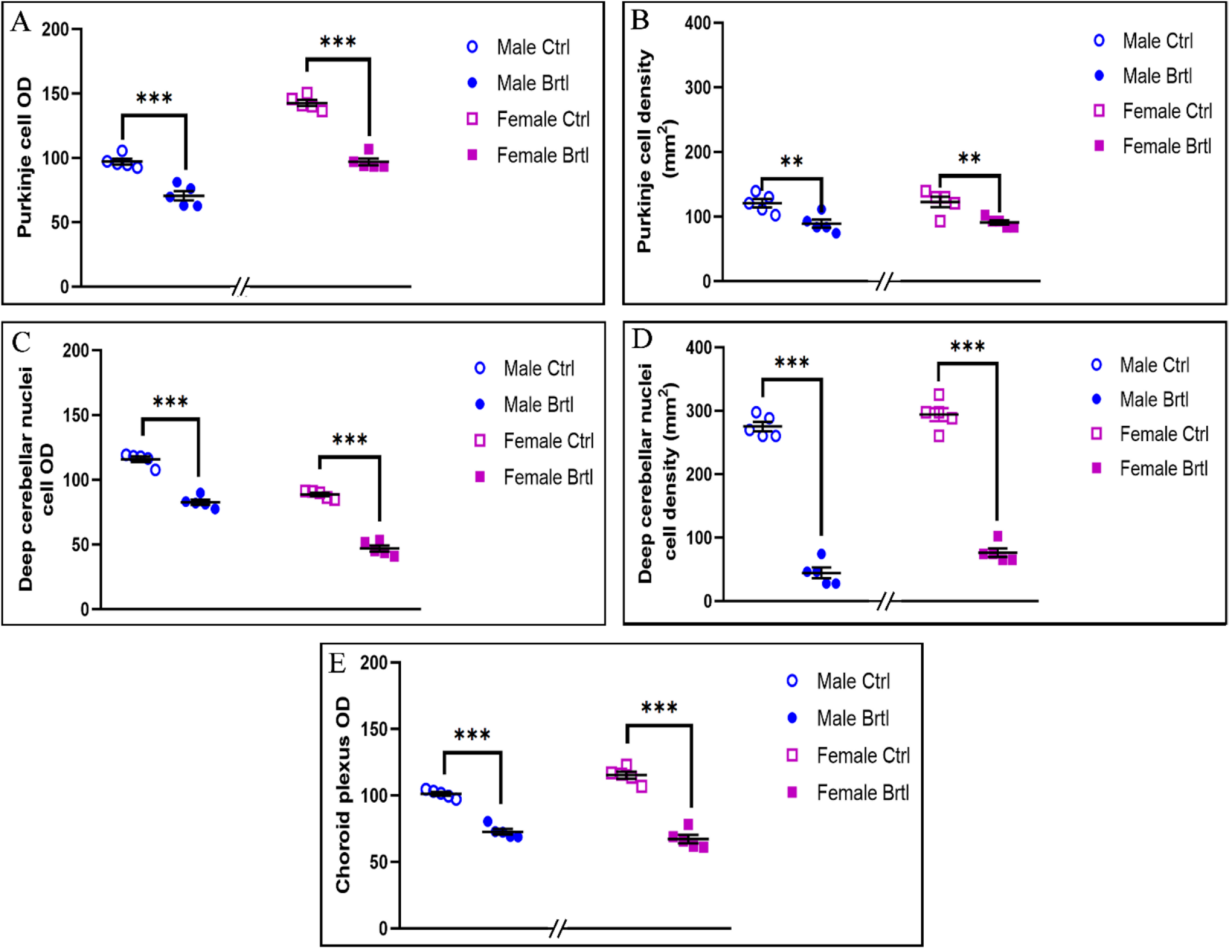
Histograms illustrating GPX4-immunopositive OD (Panels **A**, **C**, and **E**) and cell density (Panels **B** and **D**) assessed in cerebellar layers and CP of control and Brtl mice. *p*-values: (**) *p* < 0.01 and (***) *p* < 0.001

GPX4 plays a crucial role in cellular protection against oxidative stress by using GSH as an electron donor to reduce toxic lipid-OOH into non-toxic lipid-OH, preventing the breakdown of lipid-OOHs into reactive compounds and reducing oxidative stress levels [36]. Immunohistochemical reactions for GPX4 demonstrated a strong reduction of immunolabeling in all the evaluated cerebellar areas of *Brtl* mice (Fig. 9). Specifically, a significant reduction in GPX4 immunopositive OD and cell density was observed in Purkinje cells (Fig. 10, Panel A, Table S20, and Panel B, Table S21, for immunopositive OD and cell density, respectively) and in DCN cells (Fig. 10, Panel C, Table S22, and Panel D, Table S23, for immunopositive OD and cell density, respectively) of both male and female *Brtl* animals compared to their respective control groups. An extremely significant reduction in GPX4 immunolabeled OD was also detected in the CP of *Brtl* mice of both sexes compared to control animals (Fig. 10, Panel E, Table S24). However, while the OD measured in the Purkinje cells of control female mice was higher compared to the control males, this trend was reversed when measuring the immunopositive OD in the deep cerebellar nuclei cells of the two control groups, where females showed lower levels of GPX4 immunolabeled OD compared to males group (data not shown).

**Fig. 11:**
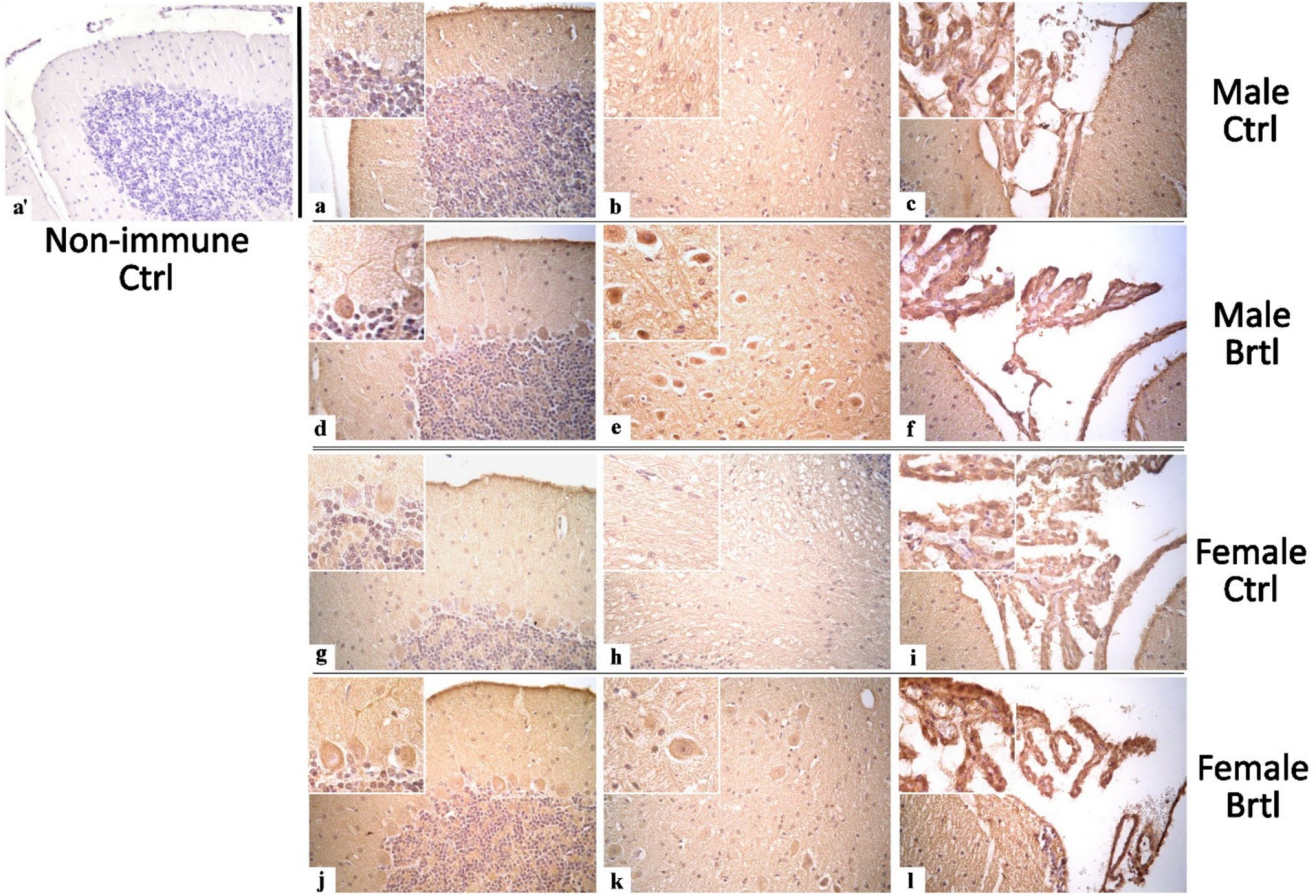
Micrographs depicting immunohistochemical reaction for NRF2 in the cerebellum from control (**a**–**c** and **g**–**i** for male and female respectively) and Brtl animals (**d**–**f** and **j**–**l** for male and female respectively). Non-immune control (**a’**). Magnification: × 40 (**a’** and **a**–**l**); × 60 (inserts)

**Fig. 12:**
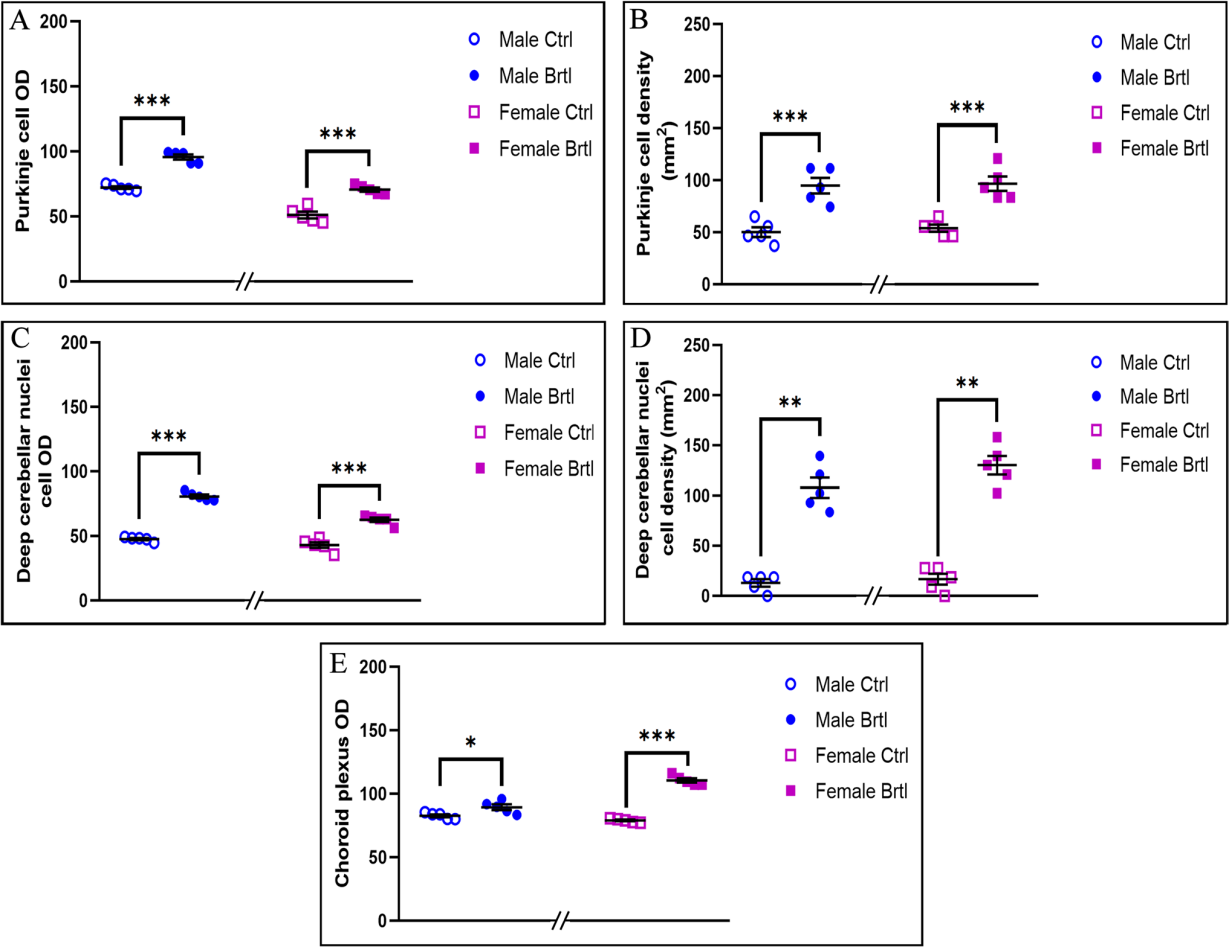
Histograms illustrating NRF2-immunopositive OD (Panels **A**, **C,** and **E**) and cell density (Panels **B** and **D**) assessed in cerebellar layers and CP of control and Brtl mice. *p*-values: (*) *p* < 0.05, (**) *p* < 0.01, and (***) *p* < 0.001

Under oxidative stress, NRF2 regulates an extensive panel of antioxidant molecules involved in the regulation of the redox state [37]. In the cerebellum of *Brtl* mice, a significant increase of NRF2 immunopositivity was highlighted after the immunohistochemical reaction (Fig. 11). In particular, the NRF2 immunopositive OD and cell density appeared statistically significant increase in the Purkinje cells (Fig. 12, Panel A, Table S25, and Panel B, Table S26, for immunopositive OD and cell density, respectively) and the cells of deep cerebellar nuclei (Fig. 12, Panel C, Table S27, and Panel D, Table S28, for immunopositive OD and cell density, respectively) of both male and female *Brtl* mice, compared to the control groups. Similarly, a significant increase in NRF2 immunopositive OD, though to a different extent, was observed in the endothelial cells of the CP of transgenic mice of both sexes (Fig. 12, Panel E, Table S29).

### TEM Ultrastructural Evaluation

**Fig. 13:**
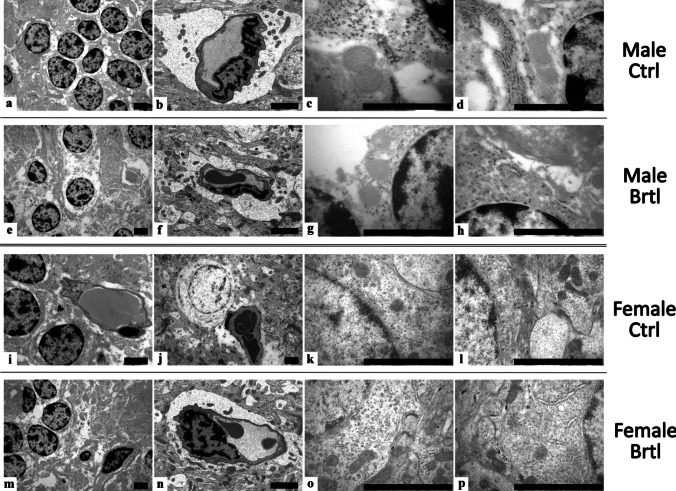
Representative cerebellar specimens investigated by transmission electron microscopy TEM from control (**a**–**d** and **i**–**l** for male and female respectively) and *Brtl* animals (**e**–**h** and **m**–**p** for male and female respectively). Scale bars: 2 µm

**Fig. 14:**
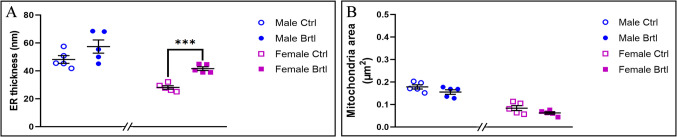
Histograms illustrating ER thickness and mitochondria area (Panels **A** and **B**, respectively) measured in cerebellar cells of control and *Brtl* mice. *p*-values: (***) *p* < 0.001

Electron microscopy analysis of the cerebellar tissue samples revealed the ultrastructural organization of different cell types in the cerebellar cortex of control and *Brtl* mice (Fig. 13). The study showed a well-preserved ultrastructural morphology of both Granule and Purkinje cells in all the experimental groups evaluated. Additionally, no collagen fiber deposits were observed in the cells of the cerebellar layers examined, in any of the groups considered. Moreover, cellular organelles displayed a physiological ultrastructural morphology, e.g., a well-preserved nuclear and Golgi apparatus ultrastructure. However, *Brtl* neurons displayed a certain degree of mitochondrial and endoplasmatic reticulum (ER) alteration. In particular, a slight but non-significant increase in ER thickness was observed in male *Brtl* animals compared to controls. However, this increase in ER thickness becomes significant when comparing control and mutant females (Fig. 14, Panel A, Table S30). On the other hand, a slight reduction in mitochondrial area was observed in both mutant groups when compared to their respective control groups (Fig. 14, Panel B, Table S31). It should also be noted that both ER thickness and mitochondrial size were larger in the male control group compared to the female control animals. Contrarily, in *Brtl* animals of both sexes, a reduction in the thickness of the vessel walls compared to control mice was also observed (data not shown).

## Discussion

The unifying hallmark of osteogenesis imperfect (OI) is the direct involvement of collagen I, a molecule involved in the regulation of the structural framework of ECM and therefore a player in modulating biophysical properties of the ECM [38]. OI is often referred to as a collagen-related disorder caused by defects in collagen structure, folding, post-translational modification, and processing which finally result in defects in bone stability and integrity. In the CNS, the ECM plays an active role in modulating homeostasis and regulating the ability to adapt and remodel in response to stress, thereby helping to preserve the balance between neuronal, glial, and vascular components [14].

Therefore, type I collagen plays a crucial role in the bidirectional crosstalk between the matrix and nervous and glial cells. Parallelly, previous literature data reported both direct and indirect effects of REDOX state on collagen levels. In particular, increased oxidative stress activates matrix metalloproteinases (MMPs), which leads to a subsequent decrease in fibrillar collagen synthesis [20, 39]. On the other hand, hydroxyl radicals, in the presence of oxygen, appear to directly cleave collagen into small peptides, resulting in an alteration of the molecule’s functionality [40]. In this regard, in the present work, morphological and ultrastructural cerebellum alterations, as well as changes in the expression level of specific markers of the oxidative stress pathway, i.e., COX4, SOD2, GPX4, and NRF2, were evaluated in a central nervous system region playing a key role in motor coordination, the cerebellum. A wide range of microscopy techniques, starting from bright field microscopy to polarized light and transmission electron microscopy was employed.

### Cytoarchitectural Alterations of the Cerebellar Cortex

The results collected have demonstrated a strong alteration in cerebellar cortex cytoarchitecture, characterized by a significant cell density depletion detected in both the molecular and granular layers in the *Brtl* animals. This morphological alteration may stem from improper development of the cerebellar cortex, due to abnormal migration of granule cells from the primitive external granular layer to the IGL, as well as subsequent alteration in the migratory processes driving the movements of different cell populations involved in the ML formation. Indeed, microarray analysis at different developmental stages has shown that collagen type I, similarly to laminin, is transiently expressed in the developing cerebellum, with expression levels decreasing by the end of the third postnatal week, coinciding with cerebellar maturation. The temporal overlap of collagen and laminin expression in the developing cerebellum suggests a possible functional interaction between these two ECM molecules during cerebellar development [13]. Therefore, we may suppose that alterations in neuronal migration processes during cerebellar development could disrupt proper circuit formation, impairing normal neuronal function. This could lead to increased cerebellar oxidative stress levels, which in turn may exacerbate neuronal damage and cellular depletion through a feedback mechanism [41–44].

### Reduction of Thick Collagen

In mutated animals of both sexes, a significant reduction in collagen levels was observed in both the superficial layers of the cerebellum, i.e., the cerebellar meninges, and in deeper structures, i.e., the choroid plexus. A more detailed investigation using polarized light microscopy revealed that the greatest impact on collagen reduction involved thick fibrils. In contrast, thin fibrils in male mice were less susceptible to this collagen depletion, highlighting not only a reduction in the total expression levels of this molecule but also a probable impairment of its maturation mechanisms, with a different impact between the genders and reduced susceptibility in males. Collagen is a fundamental component of meningeal cells, responsible for the expression and secretion of high levels of fibrous collagen in the ECM of the meningeal layers [14]. The changes in collagen levels detected in the meninges of *Brtl* mice corroborate the hypothesis of a possible alteration in the migratory processes of cerebellar cells, considering the multiple key roles that these protective layers play in the CNS, ranging from cell migration and survival regulation to vascularization and neural progenitor generation [45]. Additionally, it is known that collagen type I fibers are present in the stroma just below the epithelial cells of the choroid plexus [46]. Therefore, a reduction in collagen levels or an alteration in its maturation process in this region of the CNS could lead to functional changes in the choroid plexus, affecting its blood-cerebrospinal fluid barrier function [47].

#### Dysregulation of Oxidative Stress Pathway

Recent studies have demonstrated how mutant collagen accumulation in the endoplasmic reticulum during collagen biosynthesis leads to ER stress [48–50]. Moreover, it is now well-established that communication between the ER and mitochondria (ER-MITO contacts) is essential for coordinating cellular responses [51, 52]. Therefore, it is reasonable to hypothesize that ER stress may impact mitochondrial function, potentially leading to mitochondrial dysfunction. This could disrupt cellular energy metabolism, increase reactive oxygen species production, damage cellular components, and ultimately contribute to neuronal loss [53, 54]. In line with this hypothesis, our analyses of the oxidative stress pathway revealed widespread alteration in the REDOX balance of different areas, both superficial and deep, in the cerebellum of mutated animals, as well as differing involvement of this pathway between the two genders examined. Specifically, although a similar reduction in COX4 expression levels is observed in the cerebellar cortex of both *Brtl* groups compared to control mice, the expression levels of this mitochondrial respiratory chain enzyme in the deeper regions of the cerebellum show different patterns between males and females. In particular, there is a more pronounced reduction in COX4 expression in the deep cerebellar nuclei of females, while males show a more significant impairment in the choroid plexus. COX4, a key regulatory subunit of mammalian cytochrome c oxidase, plays a crucial role in regulating oxidative phosphorylation and contributes to chemoresistance and cancer progression [55, 56]. The observed reduction in COX4 levels could indicate mitochondrial impairment due to partial depletion of cytochrome c oxidase subunit 4, leading to alterations in intracellular ROS levels and disruptions in synaptic function and neuronal plasticity [57], resulting in impaired neurotransmission and consequent motor deficits [58]. Superoxide dismutase 2 is one of the main antioxidant enzymes in the inner mitochondrial matrix involved in regulating intracellular ROS levels through its direct action on superoxide, thus representing a first line of defense against potential mitochondrial oxidative damage resulting from increased oxidative stress, by blocking mitochondrial ROS toxicity processes [59, 60]. In this context, the data collected show a significant increase in the expression levels of this antioxidant enzyme across all cerebellar regions investigated in our OI murine model, possibly indicating a strong involvement of this enzyme in attempting to restore physiological ROS levels in the disease. This antioxidant effect seems to be more prominent in females compared to males, suggesting either an increased ROS production in females leading to greater enzyme involvement, or a more efficient ROS scavenging mechanism in females than in males. Further studies aimed at evaluating ROS levels, possibly through the use of specific tools for the evaluation of intracellular reactive oxygen species levels, could provide better insight into this regulatory mechanism of the oxidative stress pathway. In parallel, Glutathione peroxidase 4 uses glutathione as a substrate, which leads to the reduction of cellular lipid peroxides to their corresponding alcohols [61]. Therefore, the reduced expression levels of GPX4 observed in the evaluated cerebellar regions of *Brtl* mice, similarly in both sexes, could lead to an increased risk of cell death due to the accumulation of lipid peroxides in the cells of these cortical and subcortical cerebellar areas by ferroptosis activation, a mechanism of cell death associated with GPX4 inactivation that leads to lipid oxidative stress [36]. Similarly, NRF2 plays a key role in the defense against reactive oxygen species and the regulation of cellular REDOX levels [62], by controlling the expression of antioxidant genes and protecting tissues from oxidative damage [63]. Under physiological conditions, this nuclear factor resides in the cytoplasm and undergoes degradation via a ubiquitination mechanism regulated by its interaction with kelch-like ECH-associated protein 1 (KEAP1). However, under oxidative stress conditions, the interaction with KEAP1 is inhibited, allowing NRF2 to translocate into the nucleus and regulate the transcription of genes involved in oxidative stress regulation [64]. Therefore, the increased cytoplasmic expression levels of NRF2 observed in all the investigated cerebellar regions of *Brtl* male and female mice may represent the activation of a stress response mechanism aimed at enhancing the cellular antioxidant response, by increasing the cytoplasmic availability of NRF2 through downregulation of its ubiquitination-mediated degradation. However, to better assess the involvement of oxidant and antioxidant agents in the proposed study, it would be useful to evaluate the expression levels of specific antagonists of the REDOX mechanism. For instance, studying the expression levels of KEAP1 could further explain the cytoplasmic increase of NRF2 and help investigate the cellular degradation pathway of this factor.

### Ultrastructural Alterations

Transmission electron microscopy analyses revealed alterations in ER thickness and mitochondrial area. Specifically, *Brtl* mice of both sexes exhibited ER thickening and a reduction in mitochondrial area compared to their respective control groups. A mild alteration in ER structure was observed in male OI mice compared to their controls, while this alteration was much more pronounced in female OI mice relative to female controls. Similarly, a slight decrease in mitochondrial area was detected in *Brtl* animals compared to controls. These ultrastructural differences in the cerebella of male and female OI mice suggest increased cellular stress due to an imbalanced redox environment. In particular, the ER alterations observed in mutant animals indicate translational stress, while the reduction in mitochondrial area may reflect cellular dysfunction [65–69]. Both phenomena are likely interconnected and associated with the increased oxidative damage detected in the *Brtl* groups [70, 71].

## Conclusions

The morphological and ultrastructural changes observed in the cerebellum, along with the increased oxidative stress detected in our study in different regions and cell populations of this CNS area, could suggest a link between the motor limitations of OI murine model—due to compromised bone tissue—and a potential posture, coordination and balance deficit linked to a CNS damage derived by increased oxidative stress and activation of specific mechanisms of regulated cell death, both driven by a common factor: mutated forms of collagen I. Further studies are needed to investigate potential compensatory effects in collagen expression levels, which may be linked to the regulation of the healthy collagen I allele in the OI cerebellum. This could be explored using molecular and genetic techniques such as Western blot and qPCR, as well as specific antibodies in immunohistochemistry, taking into account that the collagen levels measured by Picrosirius Red staining may reflect not only collagen I expression but also other collagen isoforms, e.g., collagen IV. Additionally, further research is required to better evaluate collagen fibrils in transmission electron microscopy in cerebellar regions where they appear to be most expressed, such as the meninges and choroid plexus, in order to obtain more reliable data on fibril diameter compared to PSR-stained fibrils. Equally interesting would be comparing the observed differences between male and female *Brtl* mice and further investigating the potential impact of the observed alterations in the murine model, possibly through behavioral in vivo studies and subsequent clinical trials; the research conducted to better understand the involvement of this mutated collagen isoform in the CNS opens new perspectives on the role of central nervous system in OI. In conclusion, this study opens new horizons for potential strategies in managing patients with OI, such as the possibility of designing more personalized therapies to enhance therapeutic approaches and improve patient quality of life, possibly by the restoring of physiological redox state, mitigating the osteogenesis imperfecta pathology.

## Supplementary Information

Below is the link to the electronic supplementary material.

Supplementary Material 1 (DOCX 47.2 KB)

## Data Availability

Data is provided within the manuscript or supplementary information files.
