# Supplementary material for: Osteogenesis Imperfecta: A Look into the Cerebellum of the Brtl Murine Model
Source: Mol Neurobiol. 2025 Jul 22;62(12):15609–27. doi: 10.1007/s12035-025-05205-9 (PMC12559057; doi:10.1007/s12035-025-05205-9)
Supplement: Supplementary file 1 — Supplementary Material 1 (DOCX 47.2 KB) [file 12035_2025_5205_MOESM1_ESM.docx]

**Table S1.** Statistical analysis for the quantitative valuation of ML cell density (n/mm^2^); *p<0.05.

| Experimental group |  | Experimental group | *p*-value |
| --- | --- | --- | --- |
| Male Ctrl  (3358.90 ± 635.47) | *vs* | Male Brtl  (1452,50 ± 222.37) | * |
| Female Ctrl  (3086.56 ± 390.46) | *vs* | Female Brtl  (172.84 ± 333.55 ) | * |

**Table S2.** Statistical analysis for the quantitative valuation of IGL cell density (n/mm^2^); *p<0.05, **p<0.01.

| Experimental group |  | Experimental group | *p*-value |
| --- | --- | --- | --- |
| Male Ctrl  (22876.86 ± 891.78) | *vs* | Male Brtl  (18156.23 ± 861.23) | ** |
| Female Ctrl  (25691.07 ± 1162.57) | *vs* | Female Brtl  (21152.01 ± 1041.02) | * |

**Table S3.** Statistical analysis for the quantitative valuation of bright-field PSR OD measurements in meninges; **p<0.01, ***p<0.001.

| Experimental group |  | Experimental group | *p*-value |
| --- | --- | --- | --- |
| Male Ctrl  (91.58 ± 1.6) | *vs* | Male Brtl  (67.5 ± 1.89) | *** |
| Female Ctrl  (89.32 ± 1.9) | *vs* | Female Brtl  (80.25 ± 1.77) | ** |

**Table S4.** Statistical analysis for the quantitative valuation of bright-field PSR OD measurements in CP; ***p<0.001.

| Experimental group |  | Experimental group | *p*-value |
| --- | --- | --- | --- |
| Male Ctrl  (115.53 ± 2.89) | *vs* | Male Brtl  (67 ± 0.82) | *** |
| Female Ctrl  (112.99 ± 1.82) | *vs* | Female Brtl  (59.42 ± 3.51) | *** |

**Table S5.** Statistical analysis for the quantitative valuation of polarized light PSR mature collagen OD measurements in meninges; ***p<0.001.

| Experimental group |  | Experimental group | *p*-value |
| --- | --- | --- | --- |
| Male Ctrl  (121.18 ± 5.45) | *vs* | Male Brtl  (48.56 ± 6.05) | *** |
| Female Ctrl  (121.61 ± 7.83) | *vs* | Female Brtl  (30.48 ± 5.52) | *** |

**Table S6.** Statistical analysis for the quantitative valuation of polarized light PSR immature collagen OD measurements in meninges; ***p<0.001.

| Experimental group |  | Experimental group | *p*-value |
| --- | --- | --- | --- |
| Male Ctrl  (66.1 ± 2.48) | *vs* | Male Brtl  (48.02 ± 2.37) | ***** |
| Female Ctrl  (66.12 ± 2.83) | vs | Female Brtl  (32.68 ± 2.36) | *** |

**Table S7.** Statistical analysis for the quantitative valuation of polarized light PSR mature collagen OD measurements in CP; **p<0.01, ***p<0.001.

| Experimental group |  | Experimental group | *p*-value |
| --- | --- | --- | --- |
| Male Ctrl  (73.81 ± 7.09) | *vs* | Male Brtl  (44.85 ± 4.81) | ** |
| Female Ctrl  (59.14 ± 3.76) | *vs* | Female Brtl  (23.62 ± 4.78) | *** |

**Table S8.** Statistical analysis for the quantitative valuation of polarized light PSR immature collagen OD measurements in CP; *ns*: not significant, **p<0.001.

| Experimental group |  | Experimental group | *p*-value |
| --- | --- | --- | --- |
| Male Ctrl  (38.64 ± 4.16) | *vs* | Male Brtl  (29.42 ± 2.97) | *ns* |
| Female Ctrl  (35.73 ± 4.01) | *vs* | Female Brtl  (18.72 ± 2.55) | ** |

**Table S9.** Statistical analysis for the quantitative valuation of COX4-immunopositive OD measurements in PC; ***p<0.001.

| Experimental group |  | Experimental group | *p*-value |
| --- | --- | --- | --- |
| Male Ctrl  (125.28 ± 3.34) | *vs* | Male Brtl  (68.36 ± 3.8) | *** |
| Female Ctrl  (132.35 ± 1.92) | *vs* | Female Brtl  (104.13 ± 2.14) | *** |

**Table S10.** Statistical analysis for the quantitative valuation of COX4-immunopositive PC density (n/mm^2^); *ns*: not significant.

| Experimental group |  | Experimental group | *p*-value |
| --- | --- | --- | --- |
| Male Ctrl  (68.85 ± 4.74) | *vs* | Male Brtl  (50.24 ± 4.74) | *ns* |
| Female Ctrl  (70.71 ± 2.28) | *vs* | Female Brtl  (66.99 ± 1.86) | *ns* |

**Table S11.** Statistical analysis for the quantitative valuation of COX4-immunopositive OD measurements in mossy fiber rosettes; ***p<0.001.

| Experimental group |  | Experimental group | *p*-value |
| --- | --- | --- | --- |
| Male Ctrl  (72.84 ± 2.79) | *vs* | Male Brtl  (53.62 ± 1.52) | *** |
| Female Ctrl  (91.34 ± 4.41) | *vs* | Female Brtl  (57.45 ± 1.25) | *** |

**Table S12.** Statistical analysis for the quantitative valuation of COX4-immunopositive OD measurements in deep cerebellar nuclei cells; *p<0.05, ***p<0.001.

| Experimental group |  | Experimental group | *p*-value |
| --- | --- | --- | --- |
| Male Ctrl  (135.77 ± 3.4) | *vs* | Male Brtl  (118.39 ± 5.49) | * |
| Female Ctrl  (182.16 ± 5.66) | *vs* | Female Brtl  (108.1 ± 2.27) | *** |

**Table S13.** Statistical analysis for the quantitative valuation of COX4-immunopositive deep cerebellar nuclei cells density (n/mm^2^); *p<0.05, **p<0.01.

| Experimental group |  | Experimental group | *p*-value |
| --- | --- | --- | --- |
| Male Ctrl  (199.10 ± 13.99) | *vs* | Male Brtl  (135.84 ± 12.69) | * |
| Female Ctrl  (210.27 ± 12.34) | *vs* | Female Brtl  (130.25 ± 12.13) | ** |

**Table S14.** Statistical analysis for the quantitative valuation of COX4-immunopositive OD measurements in CP; *ns*: not significant, ***p<0.001.

| Experimental group |  | Experimental group | *p*-value |
| --- | --- | --- | --- |
| Male Ctrl  (123.67 ± 4.49) | *vs* | Male Brtl  (76.71 ± 4.5) | ** |
| Female Ctrl  (110.29 ± 1.92) | *vs* | Female Brtl  (105.42 ± 2.05) | *ns* |

**Table S15.** Statistical analysis for the quantitative valuation of SOD2-immunopositive OD measurements in PC; ***p<0.001.

| Experimental group |  | Experimental group | *p*-value |
| --- | --- | --- | --- |
| Male Ctrl  (41.24 ± 0.8) | *vs* | Male Brtl  (60.19 ± 2.03) | *** |
| Female Ctrl  (37.24 ± 2.88) | *vs* | Female Brtl  (68.89 ± 4.07) | *** |

**Table S16.** Statistical analysis for the quantitative valuation of SOD2-immunopositive PC density (n/mm^2^); **p<0.01.

| Experimental group |  | Experimental group | *p*-value |
| --- | --- | --- | --- |
| Male Ctrl  (9.30 ± 2.94) | *vs* | Male Brtl  (40.94 ± 3.72) | ** |
| Female Ctrl  (20.47 ± 3.48) | *vs* | Female Brtl  (85.60 ± 5.43) | ** |

**Table S17.** Statistical analysis for the quantitative valuation of SOD2-immunopositive OD measurements in deep cerebellar nuclei cells; ***p<0.001.

| Experimental group |  | Experimental group | *p*-value |
| --- | --- | --- | --- |
| Male Ctrl  (20.69 ± 0.71) | *vs* | Male Brtl  (39.37 ± 0.74) | *** |
| Female Ctrl  (21.73 ± 0.83) | *vs* | Female Brtl  (49.51 ± 3.52) | ** |

**Table S18.** Statistical analysis for the quantitative valuation of SOD2-immunopositive deep cerebellar nuclei cells density (n/mm^2^); **p<0.01.

| Experimental group |  | Experimental group | *p*-value |
| --- | --- | --- | --- |
| Male Ctrl  (3.72 ± 2.28) | *vs* | Male Brtl  (55.82 ± 4.16) | ** |
| Female Ctrl  (9.30 ± 4.16) | *vs* | Female Brtl  (135.84 ± 12.69) | ** |

**Table S19.** Statistical analysis for the quantitative valuation of SOD2-immunopositive OD measurements in CP; *ns*: not significant.

| Experimental group |  | Experimental group | *p*-value |
| --- | --- | --- | --- |
| Male Ctrl  (26.72 ± 0.41) | *vs* | Male Brtl  (27.08 ± 0.19) | *ns* |
| Female Ctrl  (27.35 ± 0.33) | *vs* | Female Brtl  (27.92 ± 0.24) | *ns* |

**Table S20.** Statistical analysis for the quantitative valuation of GPX4-immunopositive OD measurements in PC; ***p<0.001.

| Experimental group |  | Experimental group | *p*-value |
| --- | --- | --- | --- |
| Male Ctrl  (97.18 ± 2.23) | *vs* | Male Brtl  (70.63 ± 3.63) | *** |
| Female Ctrl  (142.74 ± 2.39) | *vs* | Female Brtl  (97.01 ± 2.58) | *** |

**Table S21.** Statistical analysis for the quantitative valuation of GPX4-immunopositive PC density (n/mm^2^); **p<0.01.

| Experimental group |  | Experimental group | *p*-value |
| --- | --- | --- | --- |
| Male Ctrl  (120.95 ± 6.58) | *vs* | Male Brtl  (89.32 ± 6.31) | ** |
| Female Ctrl  (122.81 ± 8.00) | *vs* | Female Brtl  (91.18 ± 3.48) | ** |

**Table S22.** Statistical analysis for the quantitative valuation of GPX4-immunopositive OD measurements in deep cerebellar nuclei cells; ***p<0.001.

| Experimental group |  | Experimental group | *p*-value |
| --- | --- | --- | --- |
| Male Ctrl  (115.98 ± 2.12) | *vs* | Male Brtl  (82.79 ± 2.02) | *** |
| Female Ctrl  (88.77 ± 1.32) | *vs* | Female Brtl  (47.07 ± 2.37) | *** |

**Table S23.** Statistical analysis for the quantitative valuation of GPX4-immunopositive deep cerebellar nuclei cells density (n/mm^2^); ***p<0.001.

| Experimental group |  | Experimental group | *p*-value |
| --- | --- | --- | --- |
| Male Ctrl  (275.40 ± 7.56) | *vs* | Male Brtl  (44.66 ± 8.53) | *** |
| Female Ctrl  (294.00 ± 10.44) | *vs* | Female Brtl  (76.29 ± 6.84) | *** |

**Table S24.** Statistical analysis for the quantitative valuation of GPX4-immunopositive OD measurements in CP; ***p<0.001.

| Experimental group |  | Experimental group | *p*-value |
| --- | --- | --- | --- |
| Male Ctrl  (101.3 ± 1.32) | *vs* | Male Brtl  (72.8 ± 2.11) | *** |
| Female Ctrl  (115.34 ± 2.6) | *vs* | Female Brtl  (67.26 ± 3.1) | *** |

**Table S25.** Statistical analysis for the quantitative valuation of NRF2-immunopositive OD measurements in PC; ***p<0.001.

| Experimental group |  | Experimental group | *p*-value |
| --- | --- | --- | --- |
| Male Ctrl  (72.26 ± 1.01) | *vs* | Male Brtl  (95.74 ± 1.96) | *** |
| Female Ctrl  (51.19 ± 2.53) | *vs* | Female Brtl  (70.67 ± 1.52) | *** |

**Table S26.** Statistical analysis for the quantitative valuation of NRF2-immunopositive PC density (n/mm^2^); ***p<0.001.

| Experimental group |  | Experimental group | *p*-value |
| --- | --- | --- | --- |
| Male Ctrl  (50.24 ± 4.74) | *vs* | Male Brtl  (94.90 ± 7.44) | *** |
| Female Ctrl  (53.96 ± 3.48) | *vs* | Female Brtl  (96.76 ± 6.96) | *** |

**Table S27.** Statistical analysis for the quantitative valuation of NRF2-immunopositive OD measurements in deep cerebellar nuclei cells; ***p<0.001.

| Experimental group |  | Experimental group | *p*-value |
| --- | --- | --- | --- |
| Male Ctrl  (47.73 ± 0.79) | *vs* | Male Brtl  (80.82 ± 1.39) | *** |
| Female Ctrl  (43.1 ± 2.16) | *vs* | Female Brtl  (62.7 ± 1.64) | *** |

**Table S28.** Statistical analysis for the quantitative valuation of NRF2-immunopositive deep cerebellar nuclei cells density (n/mm^2^); **p<0.01.

| Experimental group |  | Experimental group | *p*-value |
| --- | --- | --- | --- |
| Male Ctrl  (13.03 ± 3.72) | *vs* | Male Brtl  (107.93 ± 10.02) | ** |
| Female Ctrl  (16.75 ± 5.43) | *vs* | Female Brtl  (130.26 ± 9.30) | ** |

**Table S29.** Statistical analysis for the quantitative valuation of NRF2-immunopositive OD measurements in CP; *p<0.05, ***p<0.001.

| Experimental group |  | Experimental group | *p*-value |
| --- | --- | --- | --- |
| Male Ctrl  (82.73 ± 1.1) | *vs* | Male Brtl  (89.49 ± 2.18) | * |
| Female Ctrl  (79.02 ± 0.66) | *vs* | Female Brtl  (110.55 ± 1.7) | *** |

**Table S30.** Statistical analysis for the quantitative valuation of ER thickness (nm); *ns*: not significant, ***p<0.001.

| Experimental group |  | Experimental group | *p*-value |
| --- | --- | --- | --- |
| Male Ctrl  (48.2 ± 2.74) | *vs* | Male Brtl  (57.46 ± 4.72) | *ns* |
| Female Ctrl  (28.15 ± 1.24) | *vs* | Female Brtl  (41.62 ± 1.32) | *** |

**Table S31.** Statistical analysis for the quantitative valuation of mitochondria area (µm^2^); *ns*: not significant.

| Experimental group |  | Experimental group | *p*-value |
| --- | --- | --- | --- |
| Male Ctrl  (0.18 ± 0.01) | *vs* | Male Brtl  (0.16 ± 0.01) | *ns* |
| Female Ctrl  (0.08 ± 0.01) | *vs* | Female Brtl  (0.06 ± 0.01) | *ns* |
